# Supplementary material for: The discovery of novel noncoding RNAs in 50 bacterial genomes
Source: Nucleic Acids Res. 2024 Apr 22;52(9):5152–65. doi: 10.1093/nar/gkae248 (PMC11109978; doi:10.1093/nar/gkae248)
Supplement: gkae248_Supplemental_Files [file gkae248_supplemental_files.zip › Narunsky 50 Genomes RNA Motifs Files.zip!/Publication/66-P_ruminicola/Figures/LRC-66-46-NC_014033_1_IGR_161_1885310_1885608.sto.pdf]

LRC-66-46-NC_014033_1_IGR_161_1885310_1885608.sto.r2r

5′-○○○○○○○○○○●●○○○○○○○○○○○○○○○●R●●Y●●●RR●●●R●G●●AY●UU●GGGU●GY●●●UGACGUUYCU●U●GCYCG●A●●Y●●GUCACUUUGCR●UCGUAG●YR●G●YACUUU●CCCCCCUAAR●UR●C●●CGGAACGR●●CGUAGAUGAC●●CGGAACRRRY●GAAGAURACGYY●GRACRR●YC●AAA●YR●C●URG●Y●R○○○○○○○○○○○○○○○○○○○○○○○○○○○○○○○○○○
